# Health Tract, No. 262

**Published:** 1866-09

**Authors:** 


HEALTH TRACT, No. 262.

---

### THE FATAL CIGAR.

I had personally known him for thirty-five years, standing six feet four, with perfect proportions otherwise. He was a man of mark among the many passengers on the European steamer; but scarcely had the noble vessel passed Sandy Hook before the man seemed troubled. Something was discovered to be missing; not intrinsically of much value, but it could not be replaced during the voyage, and yet it was a constant need—he had left his tobacco. There could not an ounce be found on the whole ship that was not far inferior to the high brand which he had been in the habit of using. But in one of those moments in which gifted minds rise to their proper dignity, he suddenly resolved, "I'll be a slave no longer.—I shall not touch it during my two years expected absence abroad." And he did not. He occupied for a quarter of a century the highest social position in the great city of his adoption; his personal position was commanding; in his profession, he was second to none; as a writer, he was prominent. Thirty years ago I knew him as one of nature's orators. He had a majesty of presence possessed by few of the monarchs of the earth; while the courtliness of his manners and the kindness of his nature were so blended that he was the loved and revered of every circle in which he mingled.

Some unremembered time after his return, he was met by a clerical brother who after congratulating him upon his improved appearance and apparent vigorous health, asked him if he would not take a cigar.—Said he, "I have not used one in a long time, for years, in fact, but I will smoke one with you;" and it was done then and there. His old passion came upon him "like a strong man armed," and he yielded himself more and more hopelessly to it until no moment of day-light found him from under its baleful influence. The Summer of 186– found him at Saratoga. He seemed the perfection of manly beauty as to the body. But there were whisperings as to the mind. Friends delicately advised him that he needed the quiet of home. He could not think of such a thing; he greatly preferred remaining where he was. Matters became urgent. His family were telegraphed of the urgency of his retirement from public associations.

He reached home a wreck. The strong, the brilliant mind was gone. Except the members of his family and a single clergyman, no one was allowed to enter the sick-chamber. But he passed away in driveling idiocy, attendant on softening of the brain, which, with the whole nervous system had been so constantly and so long under the influence of the stimulus of tobacco, they could no longer be roused to sensation, and mind and body both gave away together.

It may admit of question which should be most pitied, the miserable victim of an unrestrained animal indulgence, dead; or the living tempter to take "The Fatal Cigar."

